# Effects of a Biocomplex Formed by Two Scaffold Biomaterials, Hydroxyapatite/Tricalcium Phosphate Ceramic and Fibrin Biopolymer, with Photobiomodulation, on Bone Repair

**DOI:** 10.3390/polym14102075

**Published:** 2022-05-19

**Authors:** Carlos Henrique Bertoni Reis, Rogerio Leone Buchaim, Karina Torres Pomini, Abdul Latif Hamzé, Isabella Vasconcelos Zattiti, Marco Antonio Hungaro Duarte, Murilo Priori Alcalde, Benedito Barraviera, Rui Seabra Ferreira Júnior, Fenelon Martinho Lima Pontes, Carlos Roberto Grandini, Adriana de Cássia Ortiz, Simone Ortiz Moura Fideles, Renata Maria de Camargo Eugênio, Geraldo Marco Rosa Junior, Daniel de Bortoli Teixeira, Eliana de Souza Bastos Mazuqueli Pereira, João Paulo Galletti Pilon, Maria Angelica Miglino, Daniela Vieira Buchaim

**Affiliations:** 1 UNIMAR Beneficent Hospital (HBU), University of Marilia (UNIMAR), Marilia 17525-160, Brazil; dr.carloshenriquereis@usp.br (C.H.B.R.); joao.pilon@abhu.com.br (J.P.G.P.); 2 Department of Biological Sciences, Bauru School of Dentistry (FOB/USP), University of São Paulo, Bauru 17012-901, Brazil; karinatp@usp.br (K.T.P.); adrianaortiz@usp.br (A.d.C.O.); simoneortiz@usp.br (S.O.M.F.); 3 Graduate Program in Anatomy of Domestic and Wild Animals, Faculty of Veterinary Medicine and Animal Science, University of São Paulo (FMVZ/USP), São Paulo 05508-270, Brazil; miglino@usp.br; 4 Postgraduate Program in Structural and Functional Interactions in Rehabilitation, Postgraduate Department, University of Marilia (UNIMAR), Marilia 17525-902, Brazil; danielteixeira@unimar.br (D.d.B.T.); elianabastos@unimar.br (E.d.S.B.M.P.); danibuchaim@alumni.usp.br (D.V.B.); 5 Medical School, University of Marilia (UNIMAR), Marilia 17525-160, Brazil; abdullhamze@hotmail.com (A.L.H.); isabella_zattiti@hotmail.com (I.V.Z.); renataeugenio@hotmail.com (R.M.d.C.E.); 6 Department of Dentistry, Endodontics and Dental Materials, Bauru School of Dentistry, University of São Paulo (FOB/USP), Bauru 17012-901, Brazil; mhungaro@fob.usp.br; 7 Department of Health Science, Unisagrado University Center, Bauru 17011-160, Brazil; murilo_alcalde@hotmail.com (M.P.A.); geraldo.junior@unisagrado.edu.br (G.M.R.J.); 8 Center for the Study of Venoms and Venomous Animals (CEVAP), São Paulo State University (Univ Estadual Paulista, UNESP), Botucatu 18610-307, Brazil; bbviera@gmail.com (B.B.); rui.seabra@unesp.br (R.S.F.J.); 9 Graduate Program in Tropical Diseases, Botucatu Medical School (FMB), São Paulo State University (UNESP–Univ Estadual Paulista), Botucatu 18618-687, Brazil; 10 Chemistry Department, Faculty of Science, São Paulo State University (UNESP–Univ Estadual Paulista), Bauru 17033-360, Brazil; fm.pontes@unesp.br; 11 Laboratório de Anelasticidade e Biomateriais, Physics Department, Faculty of Science, São Paulo State University (UNESP–Univ Estadual Paulista), Bauru 17033-360, Brazil; carlos.r.grandini@unesp.br; 12 Faculdade Ibero Americana de São Paulo, FIASP, Piraju 18810-818, Brazil; 13 Postgraduate Program in Animal Health, Production and Environment, University of Marilia (UNIMAR), Marília 17525-902, Brazil; 14 Postgraduate Program in Speech Therapy, Sao Paulo State University (UNESP—Univ Estadual Paulista), Marília 17525-900, Brazil; 15 Teaching and Research Coordination of the Medical School, University Center of Adamantina (UniFAI), Adamantina 17800-000, Brazil

**Keywords:** bone regeneration, bone repair, biomaterials, fibrin tissue adhesive, fibrin, low-level laser therapy, photobiomodulation

## Abstract

There are several treatment methods available for bone repair, although the effectiveness becomes limited in cases of large defects. The objective of this pre-clinical protocol was to evaluate the grafting of hydroxyapatite/tricalcium phosphate (BCP) ceramic biomaterial (B; QualyBone BCP^®^, QualyLive, Amadora, Portugal) together with the heterologous fibrin biopolymer (FB; CEVAP/UNESP Botucatu, Brazil) and with photobiomodulation (PBM; Laserpulse^®^, Ibramed, Amparo, Brazil) in the repair process of bone defects. Fifty-six rats were randomly divided into four groups of seven animals each: the biomaterial group (G1/B), the biomaterial plus FB group (G2/BFB); the biomaterial plus PBM group (G3/B + PBM), and the biomaterial plus FB plus PBM group (G4/BFB + PBM). After anesthesia, a critical defect was performed in the center of the rats’ parietal bones, then filled and treated according to their respective groups. The rats were euthanized at 14 and 42 postoperative days. Histomorphologically, at 42 days, the G4/BFB + PBM group showed a more advanced maturation transition, with more organized and mature bone areas forming concentric lamellae. A birefringence analysis of collagen fibers also showed a more advanced degree of maturation for the G4/BFB + PBM group. In the comparison between the groups, in the two experimental periods (14 and 42 days), in relation to the percentage of formation of new bone tissue, a significant difference was found between all groups (G1/B (5.42 ± 1.12; 21.49 ± 4.74), G2/BFB (5.00 ± 0.94; 21.77 ± 2.83), G3/B + PBM (12.65 ± 1.78; 29.29 ± 2.93), and G4/BFB + PBM (12.65 ± 2.32; 31.38 ± 2.89)). It was concluded that the use of PBM with low-level laser therapy (LLLT) positively interfered in the repair process of bone defects previously filled with the biocomplex formed by the heterologous fibrin biopolymer associated with the synthetic ceramic of hydroxyapatite and tricalcium phosphate.

## 1. Introduction

Tissue bioengineering is developing strategic research seeking new therapeutic re-sources that can be applied in the bone regeneration process, such as stem cell differentiation, graft materials, and the use of membranes. Associated with these resources, alternative therapies are sought [1,2], aiming at the rapid formation of structurally intact bone for the surrounding skeleton [3,4].

The autologous bone graft, due to its characteristics and properties inherent to the re-generation process, is considered the gold standard [5,6]. However, therapy with autoge-nous grafts has, on the other hand, some negative points such as postoperative complications and increased surgical time [7]. In this scenario, and in the face of critical bone defects, the use of biomaterials has gained space in pre-clinical research [8,9]. An ideal bone substitute must present important characteristics such as the release of growth factors that enhance bone neoformation, promote a framework, and favor a microenvironment that enhances tissue growth [10].

Within the diversity of biomaterials indicated for the surgery of dental and orthopedic grafts, for guided bone regeneration and filling dental alveoli, the compounds of hydroxyapatite and tricalcium phosphate are used because they are generally of null cytotoxicity, biocompatible, low cost and easy to produce, and osteoconductive, and, at the insertion site, these biomaterials present a good level of vascularization and bone formation [11,12,13,14]. The biomaterial hydroxyapatite/tricalcium phosphate (QualyBone BCP^®^, QualyLive, Amadora, Portugal) is a synthetic ceramic containing 75% hydroxyapatite and 25% tricalcium phosphate. It has a macroporosity that facilitates the proliferation of bone cells and neovascularization in empty spaces. This ceramic is already commercially available and used clinically in the reconstructive surgery of bone lesions [15].

However, the joint use of particulate biomaterials with other biological scaffolds can also be performed. This association favors the insertion and permanence of the material in the graft receptor site, in addition to allowing better functional mechanisms of tissue regeneration [16]. Thus, some bioproducts can be indicated for this purpose, among them, sealants or fibrin adhesives. These are used in surgery as hemostatic agents and inducers of the healing process [17]. Being identified as active biological scaffolds, they play an important role as a structure and/or anchor for cell fixation and growth [18,19,20,21].

The biological principles of fibrin patches mimic the end of the coagulation cascade, which normally occurs in the human body [18,22,23]. The heterologous fibrin sealant (HFS) derived from snake venom was developed by the Center for the Study of Venoms and Venomous Animals (CEVAP/UNESP, Botucatu, São Paulo, Brazil). HFS was initially used as a fibrin glue for repairing nerve injuries and healing chronic venous ulcers [24,25]. It is biocompatible and has hemostatic, sealant, and adhesive properties. It currently has a variety of clinical applications as it is able to act as a scaffold for stem cells [26,27] and as a drug delivery system [28]. Considering all the properties of this bioproduct, the name “sealant” was reconsidered, and it became known as “heterologous fibrin biopolymer” [29,30,31].

In this context, alternatives are being developed and explored with the objective of minimizing bone regeneration time and reducing the chance of possible complications resulting from the deficient consolidation process. Among them, photobiomodulation therapy (PBM) stood out for its satisfactory effects on metabolism and bone repair. This was due to its great osteogenic potential as it is a therapy that acts positively in the process of stimulating bone repair [32,33].

The photobiostimulatory effects of laser are directly related to cellular responses. When applied, their activities are accelerated, resulting in increased mitochondrial respiration and ATP synthesis. In addition, it optimizes protein synthesis, migration, and cell proliferation, and reduces the inflammatory response, decreasing edema and providing an efficient healing and bone regeneration process [34,35,36,37]. Finally, PBM therapy is a relatively low-cost, non-invasive treatment method. Despite all these advantages, there are controversies regarding the best parameters to be used to obtain an effective result in the extensive bone repair process filled with biomaterials [38].

Given the knowledge already acquired and the experiments that seek to standardize protocols and evaluate the effects of PBM therapy on the bone repair process [39], the objective of this study was to assess whether PBM, through the use of low-level laser therapy (LLLT), interferes in the repair process of bone defects filled with BCP biomaterial associated with the heterologous fibrin sealant produced by CEVAP as a scaffold, thus trying to standardize an ideal experimental protocol to be used in the bone regeneration process and aiming at future clinical trials and contributing to the scientific-technological advance of translational science.

## 2. Materials and Methods

### 2.1. Experimental Design

Fifty-six male Wistar rats (*Rattus norvegicus*), aged 12 weeks and with an average weight of 250 g, supplied by the Central Animal Facility of the University of Marília (UNIMAR, Marília, São Paulo, Brazil) were used. The animals were kept in suitable environments under a 12 h light/dark cycle with controlled temperature (23 ± 1 °C) and received balanced animal feed (Labina^®^ Purina, São Paulo, Brazil). A maximum of four animals per box were kept, and after surgery, they were allocated individually. The study was conducted in accordance with the guidelines of the Declaration of Helsinki and approved by the Ethics Committee on the Use of Animals (CEUA), University of Marília (Protocol 011/2019; 3 June 2019).

In addition, this experimental study was performed according to the ARRIVE (Animal Research: Report of in vivo Experiments) guidelines and was based on the principles of NC3Rs (National Center for Replacement, Refinement, and Reduction of Research Ani-mals). Throughout the experiment, the animals were monitored for the expression of pain, apathy, and symptoms of depression, aggression, and overexcitement, characteristics that vary in their usual behavior. Changes in gait, posture, and facial expression were also observed. Unusual behaviors such as excessive consumption of water and food, as well as possible clinical symptoms, were investigated [40].

**Figure 1:**
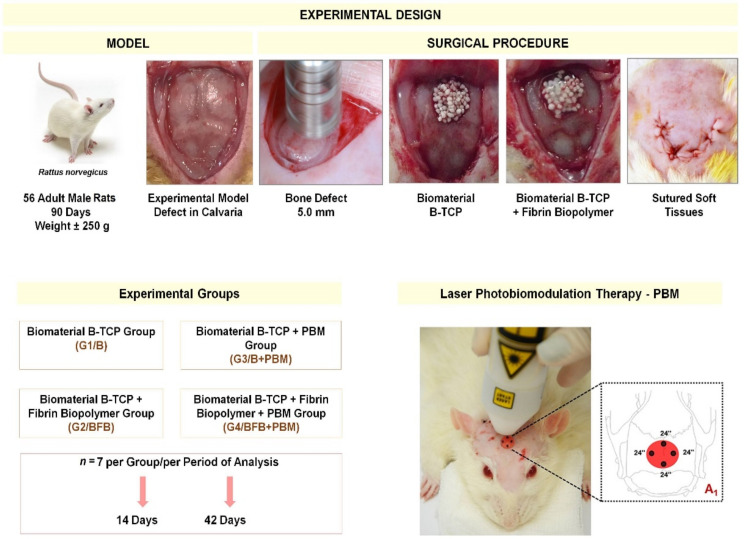
Experimental design. Animal model and inclusion criteria for 56 Wistar male rats (*Rattus norvegicus*): adults of 90 days old, weight of approximately ± 250 g; experimental model of bone defect in calvaria, exposure of parietal bones; surgical procedure: fabrication of a 5 mm diameter bone defect with a trephine drill; defect filled with biomaterial BCP (G1/B and G3/B + PBM); defect filled with biomaterial BCP and heterologous fibrin biopolymer (G2/BFB and G4/BFB + PBM); underlying soft tissue repositioned and sutured. A_1_: illustration of post-immediate photobiomodulation (PBM) therapy, followed by 3x per week until the corresponding euthanasia period for the G3/B + PBM and G4/BFB + PBM groups. Experimental periods were 14 and 42 days, with 7 animals/group/period.

The animals were randomly divided into four groups (*n* = 7 each), without predetermined inclusion or exclusion criteria, and the euthanasia periods were defined between 14 and 42 days. The groups were distributed as follows: biomaterial group (G1/B), biomaterial plus fibrin biopolymer group (G2/BFB); biomaterial plus photobiomodulation group (G3/B + PBM); and biomaterial plus fibrin bipolymer plus photobiomodulation group (G4/BFB + PBM) (Figure 1).

### 2.2. Sample Characterization–Biomaterial (BCP)

QualyBone BCP^®^ (QualyLive, Amadora, Portugal) is marketed with the European Union certification seal (CE-Conformité Européenne), which indicates its compliance with the health, safety and environmental protection standards defined for the region (Certificate ES19/86908.02). Recently, it received authorization for commercialization in Brazil by the ANVISA-Brazilian Health Regulatory Agency under No. 81634410004. The product is sterilized in its double wrapping by gamma radiation at the minimum dose of 25 kGy.

**Figure 2:**
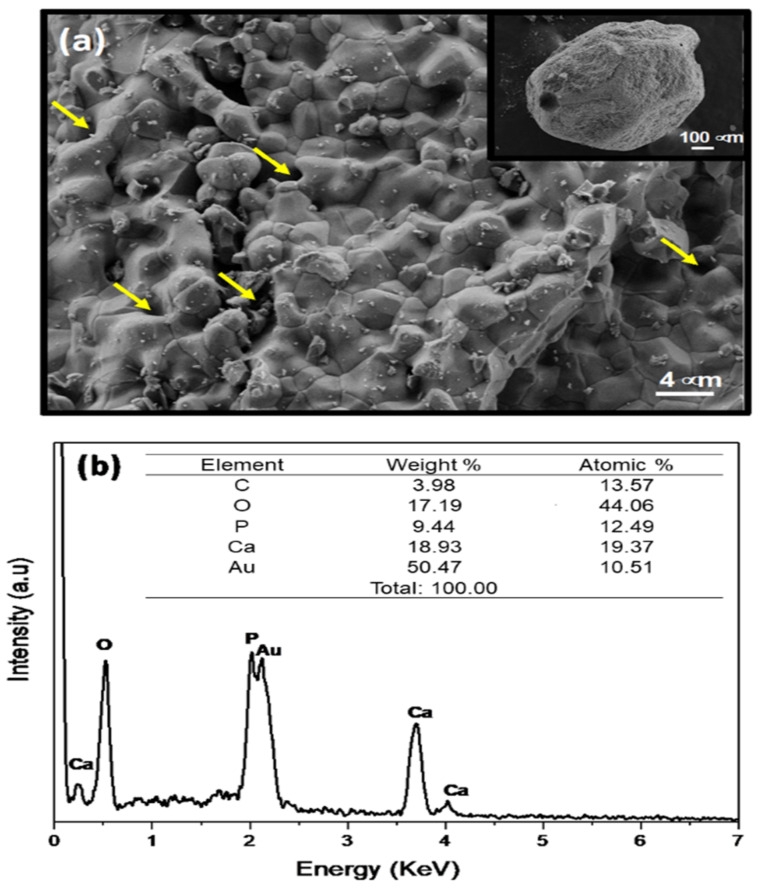
(**a**) FEG-SEM micrographs obtained for the BCP sample (yellow arrows shows porous structure). The inset reveals the aspect almost spherical particles. (**b**) EDS spectrum of the BCP sample.

**Figure 3:**
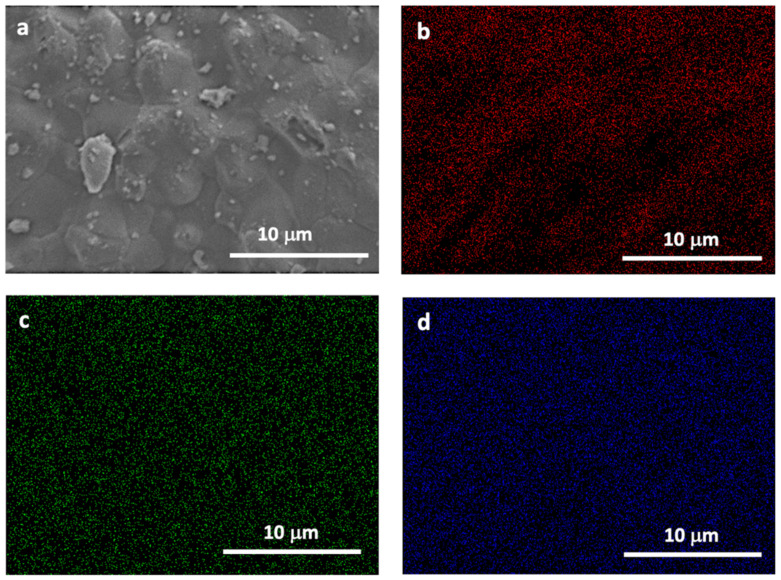
(**a**) SEM image and EDS mapping showing the (**b**) oxygen, (**c**) calcium, and (**d**) phosphor distribution of the BCP sample.

Sample morphology hydroxyapatite/tricalcium phosphate biomaterial (QualyBone BCP^®^ particles, QualyLive, Amadora, Portugal) was analyzed with a Field Emission Gun-Scanning Electron Microscope (FEG-SEM, Inspect S50, FEI, Hillsboro, OR, USA), which was operated at an accelerating voltage of 5 kV. For the mapping, energy dispersive spectroscopy (EDS) was used (INCA x-act detector, Oxford Instruments, UK), coupled to a scanning electron microscope (Figure 2 and Figure 3).

**Figure 4:**
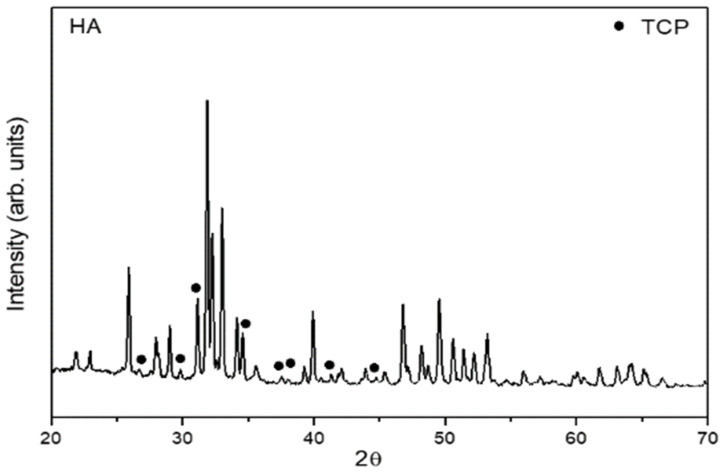
X-ray diffraction patterns of the BCP samples (75% hydroxyapatite−25% TCP).

The BCP crystal structures were investigated by analyzing the X-ray diffraction (XRD) patterns of Cu Kα radiation recorded by a diffractometer (Miniflex 600, Rigaku, Japan) in 2θ using a step size of 0.04° and an X-ray source operating at 40 KV and 15 mA with Cu-Kα radiation (Figure 4).

### 2.3. Heterologous Fibrin Biopolymer (FB)

FB derived from snake venom was kindly provided by the Center for the Study of Venoms and Venomous Animals at UNESP (CEVAP/UNESP, Botucatu, Brazil). The biopolymer is composed of three fractions separated and homogenized before its application, totaling 40 µL. The first component is fraction 1, which is composed of the thrombin-like enzyme (10 µL) added to calcium chloride diluent (10 µL). Fraction 2 is composed of fibrinogen extracted from buffalo blood (20 µL). For application in the G2/BFB and G4/BFB + PBM groups, the biopolymer components were deposited in microtubes, initially mixing fraction 1 with the diluent, adding the biomaterial, and then placing fraction 2, forming a biocomplex similar to a gelatinous substance [17,41].

### 2.4. Experimental Surgery

The animals were submitted to general anesthesia with an intramuscular injection of tiletamine hydrochloride and zolazepam hydrochloride (10 mg/kg-Telazol^®^; Fort Dodge Laboratories, IA, USA). Trichotomy was performed in the frontal-parietal bone region followed by antisepsis with a topical solution of 10% Polyvinyl Pyrrolidone Iodine PVPI (Povidine^®^ Antisseptico, Vic Pharma Ind e Comércio, São Paulo, Brazil). Then, a 4 cm half-moon incision was made with a No.15 carbon steel scalpel blade (Embramax^®^, São Paulo, Brazil) in the integument, and the periosteum was carefully detached with the aid of the syndesmatome and folded together with the other tissues, exposing the external surface of the parietal bones (Figure 1).

A circular osteotomy of 5.0 mm in diameter was performed in the center of the parietal bones (Figure 1) with the aid of a trephine drill (Neodent^®^, Curitiba, Brazil) adapted to the contra-angle (Driller^®^, São Paulo, Brazil) attached to the electric micromotor (Driller BLM 600 Baby^®^, São Paulo, Brazil) at low speed (1500 rpm). Irrigation was constant and abundant using sterile saline solution (0.9% saline solution) to prevent bone necrosis by thermal action, thus obtaining a bone fragment, without spikes, in order to preserve the integrity of the dura mater and the brain.

In the animals of the groups G1/B and G3/B + PBM, the defect was filled only with the BCP biomaterial, and in the animals of the G2/BFB and G4/BFB + PBM groups, the defects were filled with the BCP biomaterial associated with the heterologous fibrin biopolymer (FB) (Figure 1). The biomaterial was weighed on an analytical balance (MicroNal^®^ Precision Equipment, São Paulo, Brazil) to obtain a weight of approximately 0.03 mg and inserted into the defect site without exerting pressure on the brain. Tissues in the surgical area were repositioned (Figure 1), taking care that the periosteum covered the cavities, and then the integument was sutured (simple stitches) with 4–0 silk thread (Ethicon*^®^*, Johnson and Johnson Company, São Paulo, Brazil).

### 2.5. Photobiomodulation Protocol (PBM)

The G3/B + PBM and G4/BFB + PBM groups were submitted to laser treatment with galli-um-aluminum-arsenide (GaAlAs, Laserpulse IBRAMED^®^, Amparo, Brazil; registered in the ANVISA-Brazilian Health Regulatory Agency under No. 10360310030) where, in all applications, the laser beam emissions were calibrated in the device itself and previously tested to certify the dose, following the parameters described in Table 1.

### 2.6. Euthanasia and X-ray Computed Microtomography (µ-CT)

Respectively after 14 and 42 days of post-surgery, for 7 animals from each group per pe-riod, euthanasia was performed using the barbiturate (Thiopental^®^, Cristalia, Itapira, Brazil) dosage for rats (150 mg/kg) as follows: sodium thiopental 2.5%, per via intraperitoneal-IP, applied in the lower left abdominal quadrant of the animal (associated with a local anesthetic, lidocaine hydrochloride at a dose of 10 mg/kg). Then, the region of the defect of each animal was carefully removed with the aid of a dental conical surgical carbide bur mounted on a low rotation piece (Dabi Atlante^®^, Ribeirão Preto, Brazil) preserving the supraperiosteal soft tissues and fixed in 10% formalin solution in a phosphate buffer of pH 7.2 for one week for microtomographic analysis and, later, for histological processing.

The pieces were submitted to an X-ray beam scan in a computerized microtomograph SkyScan^®^ 1174v2 (Bruker-microCT, Kontich, Belgium) of the Bauru School of Dentistry, University of São Paulo (FOB/USP, Bauru, São Paulo, Brazil). The samples were placed in tubes, positioned, and fixed in the appropriate sample holder for the equipment. Then, they were rotated 360°, with a “rotation step” of 0.5 and isotropic resolution of 19.6 µm, generating a time of 41 min and 32 s per sample.

The images of each specimen were analyzed and reconstituted with the specific software 64 Bits270013 (Bruker, Kontich, Belgium) and the NRecon^®^ program (version.1.6.8.0, Sky-Scan, 2011, Bruker-microCT, Kontich, Belgium) in about 1000 to 1100 slices, according to the adopted anatomical parameters. The software Data Viewer^®^ version 1.4.4 64 bit (linear measurements of the coronal, transaxial, and sagittal axes, Bruker, Kontich, Belgium) and CTvox^®^ version 2.4.0 r868 (Bruker Micro CT, Bruker, Kontich, Belgium), were used for two-dimensional visualization.

### 2.7. Sample Collection and Histological Procedure

The pieces were subjected to demineralization in EDTA solution, a solution containing 4.13% tritiplex^®^ III (Merck KGaA, Hessen, Germany) and 0.44% sodium hydroxide (Labsynth, São Paulo, Brazil) with weekly changes of the solution for a period of approximately 40 days. Subsequently, semi-serial coronal sections were performed, considering the central region of the defect with the aid of the Leica^®^ RM2245 semi-automatic microtome (Leica Biosystems, Wetzlar, Germany). Sections 5 µm thick (six slides with four sections each) were made for hematoxylin-eosin and Masson’s trichrome staining. Two evaluators previously calibrated and blinded in relation to the groups and periods performed the constant analyses in the methodology.

### 2.8. Birefringence Analysis of Collagen Fibers (Picrosirius-Red Staining)

Sections stained with Picrosirius-red were evaluated under polarized light to determine the quality of the newly formed organic matrix during the experimental periods (14 and 42 days) of healing in the defects. Images were obtained from the defects using the higher resolution digital camera, Leica DFC 310FX (Leica Microsystems^®^, Wetzlar, Germany), connected to the confocal laser microscope, Leica DM IRBE, and the capture system LAS 4.0.0 (Leica Microsystems^®^, Heerbrugg, Switzerland).

### 2.9. Histomorphometric Analysis

In all specimens, the entire extent of the defect was considered to assess the bone repair pattern in all groups, with four semi-serial sections of the surgical bed of each defect being evaluated with an Olympus^®^ light microscope (Olympus Corporation, Tokyo, Japan).

Quantitative image analysis was performed on a computer (Core I7 Processor; Intel Corporation, Santa Clara, CA, USA) using Carl Zeiss AxioVision (Rel. 4.8.2 White Plains, NY, USA). From the semi-serial sections obtained, two more central sections of the defect with a distance between them of 300 µm were captured. The percentage of newly formed bone tissue, biomaterial, and non-mineralized tissue was calculated.

### 2.10. Statistical Analysis

The data were subjected to analysis of variance (ANOVA) to detect possible differences between groups. The ANOVA assumptions, normality of residuals, and homogeneity of variances were verified, respectively, by the Shapiro–Wilk and Bartlett tests, both at 5% probability. Subsequently, the means were compared by Tukey test at 5% probability. Within each treatment, the comparison of new bone formation, biomaterial, and non-mineralized tissue as a function of the treatment period (14 and 42 days) was evaluated using the Student’s t-test at 5% probability. All analyses were conducted using the R software (R Core Team, Vienna, Austria).

## 3. Results and Discussion

Regarding the in vivo studies, there were no complications that needed to be reported, and there was no disease or sign that strongly motivated the removal of an animal (clinical outcome).

### 3.1. Sample Characterization

The morphology of the sample, observed by FEG-SEM, is shown in Figure 2. The inset in Figure 2 shows almost spherical particles obtained for BCP. FEG-SEM provides details of a constituent structure which clearly reveals that the BCP sample mostly consists of particles submicron size of order 2 μm that are homogeneous and have a uniform distribution and high degree of packing (Figure 2a). The energy dispersive spectroscopy (EDS) spectra of the elements show peaks only for the elements (oxygen-O, phosphor-P, and calcium-Ca) (Figure 2b). In addition, the gold signal has been observed. The sample was coated with gold before SEM analysis.

The distribution of the elements in the samples was evaluated using EDS image mapping of the surfaces where red points represent oxygen, blue points represent phosphor, and green points represent calcium. A good distribution of the elements, without precipitates or aggregates, was observed in both samples (Figure 3), indicating good homogeneity.

The XRD pattern showed that the BCP samples exhibited well-defined diffraction peak characteristic of hydroxyapatite (HA, major phase), which is confirmed by the no. #09-0432 JCPDS card. In addition, the XRD pattern of the BCP samples showed diffraction peaks corresponding the minor phase (tricalcium phosphate/TCP, black circle, JCPDS card no #09-0169, Figure 4).

Grafting materials and their properties can be improved, mainly in their characteristics that lead to better tissue performance and new bone formation. A new approach increasingly studied is ion substitutions in calcium phosphate bioceramics. Ressler, et al. (2022) prepared porous composite scaffolds based on CaPs substituted by Sr^2+^, Mg^2+^, Zn^2+^, and SeO_3_^2−^ ions and chitosan by the freezing technique. The scaffolds presented a highly porous structure with very well interconnected pores, with osteogenic potential together with human mesenchymal stem cells. The findings demonstrated that ionic substitutions have a beneficial effect on cells and tissues and increased the expression of osteogenesis-related markers and increased phosphate deposits compared to scaffolds with unsubstituted CaPs [42].

### 3.2. Qualitative Analysis of Two-Dimensional Microtomographic Images

**Figure 5:**
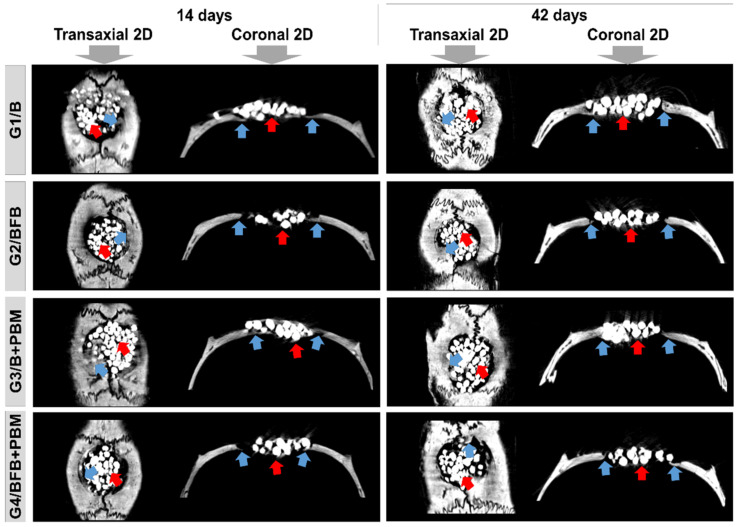
Two-dimensional (2D) reconstructed microtomographic images in transaxial and coronal sections of the bone defects in rat calvaria at 14 and 42 days, respectively. Defect filled with biomaterial (G1/B), biocomplex consisting of biomaterial plus heterologous fibrin biopolymer (G2/BFB), biomaterial and PBM (G3/B + PBM), and biocomplex consisting of biomaterial plus heterologous fibrin biopolymer and photobiomodulation with low-level laser therapy (G4/BFB + PBM). Bone formation (blue arrow) and biomaterial particles (red arrow).

At 14 days, the bidimensional (transaxial and coronal) microtomographic images showed, in all experimental groups, a centripetal pattern of bone formation, evidenced by the increased gradation of bone tissue density in gray scale in the peripheral areas of the bone defect. The biomaterial particles were surrounded by immature bone trabeculae (Figure 5).

In all groups, at 42 days, there was an increase in bone growth, but without complete closure of the defect, remaining limited to the surgical edges, and with focal areas of mineralized tissue in the G3/B + PBM and G4/BFB + PBM groups. The regions of bone remodeling were observed peripherally, relative to the difference in tissue density. The central area of the wound remained filled with biomaterial particles (Figure 5).

The intertwining of bone trabeculae with the biomaterial, observed in the initial stage of tissue repair (14 days), evidences the porous characteristic of the scaffold, which is important to favor cell proliferation and migration [43]. The process of new bone formation that occurred over the 42 days was also favored by the combination of the biomaterial with the fibrin polymer. This association of biopolymers constitutes a promising strategy to regenerate bone defects since the fibrin favors the incorporation of the biomaterial at the lesion site [44].

Tissue regeneration was also improved by PBM therapy, which stimulates bone formation and favors the integration process of the biomaterial stabilized with fibrin [44,45]. The increasing bone formation observed over time in this research was also reported in studies that used similar methodologies [20,44]. These data demonstrate that PBM may have positive effects on bone tissue, improving the quality and density of newly formed bone [44,46,47].

A study carried out with a similar biocomplex, except that the biomaterial was a bone substitute established in preclinical and clinical studies (Bio-Oss^®^ bone substitute, Geistlich Pharma AG, Wolhusen, Switzerland), with the same laser therapy protocol, showed similar results in bone neoformation increased by PBM in which the biocomplex created a favorable microenvironment for an adequate repair process as an innovative drug delivery system [44]. The phase I/II clinical trial involving the treatment of chronic venous ulcers with FB has been completed and its results were recently published [17].

### 3.3. Histomorphological Analysis

In this preclinical study, two postoperative periods were used for evaluation, 14 and 42 days. In the initial period (14 days), photobiomodulation plays an important role in the initial phases of the repair process, as it helps in the biological response by reducing the inflammatory process, decreasing pain, and creating conditions for accelerating the formation of new bone. In the final period (42 days), in non-critical defects in rats, the process would progress to complete repair of the surgical area. In the case of critical defects, which do not repair spontaneously until the end of the experiment, the evaluation of the formation of new bone is important to analyze the action of photobiomodulation and grafting materials in the evolution of the process, in addition to the amount of tissue non-mineralized material that remained inside the defect and the permanence of biomaterials at this site [48,49,50,51,52].

**Figure 6:**
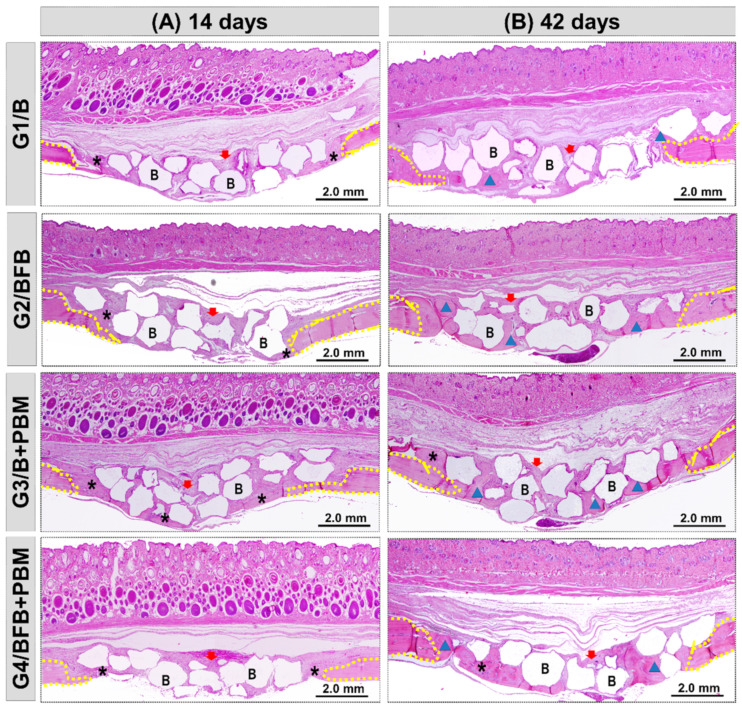
Panoramic histological views at 14 (**A**) and 42 (**B**) days in the cranial defects filled with biomaterial (G1/B), biocomplex consisting of biomaterial plus heterologous fibrin biopolymer (G2/BFB), biomaterial and PBM (G3/B + PBM), and biocomplex consisting of biomaterial plus heterologous fibrin biopolymer and photobiomodulation with low-level laser therapy (G4/BFB + PBM). Immature trabecular formation (asterisk) occurring at the edge of the defect (dashed line) and overlying the dura mater surface. Biomaterial particles (**B**) permeating the reaction connective tissue (red arrow). The transition from bone maturation to mineralized tissue (triangle), with primary bone areas (asterisk) and biomaterial particles in densely fibrous connective tissue (red arrow). HE; original magnification × 4; bar = 2 mm.

**Figure 7:**
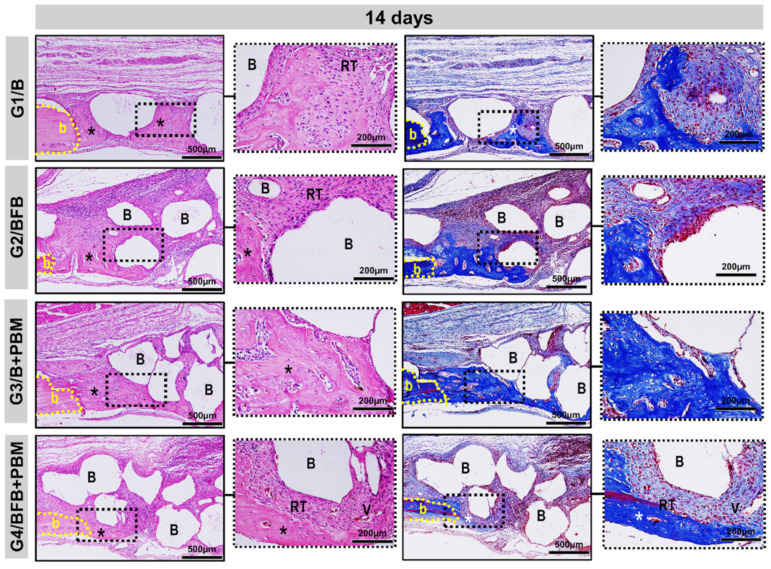
Details of the evolution of the bone repair process of the cranial defects at 14 days filled with biomaterial (G1/B), biocomplex consisting of biomaterial plus heterologous fibrin biopolymer (G2/BFB), biomaterial and PBM (G3/B + PBM), and biocomplex consisting of biomaterial plus heterologous fibrin biopolymer and photobiomodulation with low-level laser therapy (G4/BFB + PBM). The deposition of the osteoid matrix (asterisk) from the edges of the defect (b), particles of the biomaterial (B) interspersed with densely cellular reactive connective tissue (RT) and vascular budding (V). HE and Masson Trichrome; original magnification × 10; bar = 500 µm and insert, magnified images × 40; bar = 100 µm.

All the experimental groups, G1/B, G2/BFB, G3/B + PBM, and G4/BFB + PBM, exhibited peculiar characteristics at 14 days, with the area of the defect interpolated by reactive connective tissue, densely cellularized, and permeated by inflammatory cells, random arrangements of thin collagen fibers, and biomaterial particles. Bone growth described the same pattern in all defects, adjacent to peripheral regions with irregular trabecular conformation. The G4/BFB + PBM group showed a marked angiogenic response, with evident vascular sprouts (Figure 6 and Figure 7).

**Figure 8:**
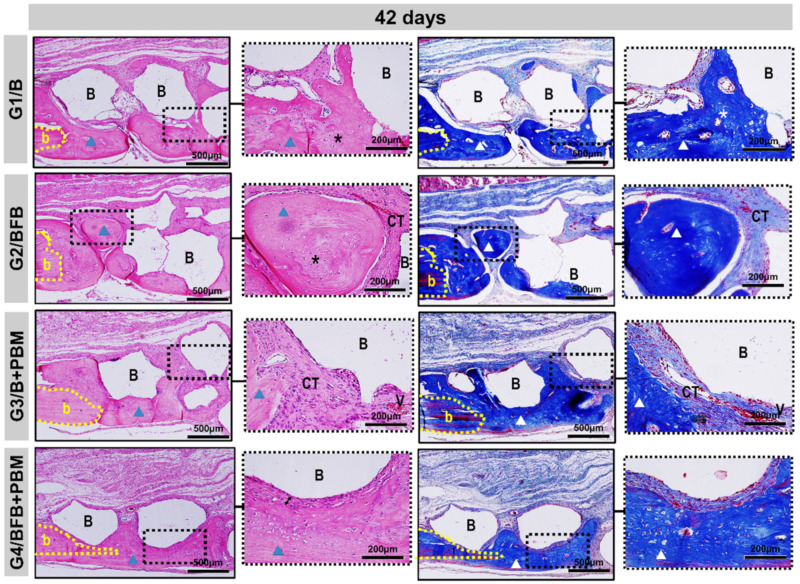
Details of the evolution of the bone repair process of the cranial defects at 42 days filled with biomaterial (G1/B), biocomplex consisting of biomaterial plus heterologous fibrin biopolymer (G2/BFB), biomaterial and PBM (G3/B + PBM), and biocomplex consisting of biomaterial plus heterologous fibrin biopolymer and photobiomodulation with low-level laser therapy (G4/BFB + PBM). Mature lamellar tissue (triangle) was restricted to the edge of the defect (b) and areas of immature bone trabeculae (asterisk) in the fibrous connective tissue (CT). Biomaterial particles (B) surrounded by thicker collagen fibers, with a fibrous interface between the particles and newly formed bone (arrow). HE and Masson Trichrome; original magnification × 10; bar = 500 µm and insert, images magnified × 40; bar = 100 µm.

At 42 days, the height of the bone remaining in the surgical region was preserved in all bone defects. In the central region, a slight invagination of overlying soft tissues was observed in the G1/B and G2/BFB groups, unlike the G3/B + PBM and G4/BFB + PBM groups. The new bone tissue showed a continuous growth, but was restricted to the edges of the defects, with mineralized bone focal areas between the biomaterial particles. The G4/BFB + PBM group exhibited a more advanced maturation transition, with more organized and mature bone areas, forming concentric lamellae, surrounded by regions of immature bone trabeculae (Figure 6 and Figure 8).

The histological data also showed positive effects regarding the combination of treatment methods in the bone regeneration process, which was already visible at 14 postoperative days. In this period of analysis, it is possible to observe the presence of vascular sprouts in the G4/BFB + PBM group, which may be due to the effects of PBM therapy on the local microcirculation [46]. In addition to PBM, the fibrin biopolymer also contributed to stimulate angiogenic factors and neovascularization, as observed in previous studies [20,44]. The analyses performed at 42 postoperative days shows that there was an advance in bone formation in all experimental groups, but without the complete closure of the defects.

The bone neoformation occurred along the edges of the defect, considering the stimuli of the microenvironment of the adjacent bone tissue, and it was limited to them, being possible to observe particles of biomaterial not yet degraded in the central region of the defect. Although all groups showed bone growth, the newly formed bone tissue in the G4/BFB + PBM group presented a more mature histological aspect, which is in agreement with studies that obtained the formation of a more organized bone tissue in the biostimulated groups [20,44,53]. These data indicate that the association of biopolymers with PBM therapy had additional effects, improving the histological characteristics of the newly formed bone tissue.

The composite of hydroxyapatite and tricalcium phosphate, selected for this experimental protocol, showed a biological response similar to several products marketed and used in bone loss restoration techniques, corroborating the properties conceptually necessary for an ideal biomaterial as they do not cause intense inflammatory reaction, they did not present encapsulation or rejection at the receptor site, and they allowed the osteoprogenitor cells adjacent to the bone defect to differentiate through the structure generated by such materials, which demonstrates osteoconduction [54,55,56,57,58].

### 3.4. Description Birefringence Analysis of Collagen Fibers

**Figure 9:**
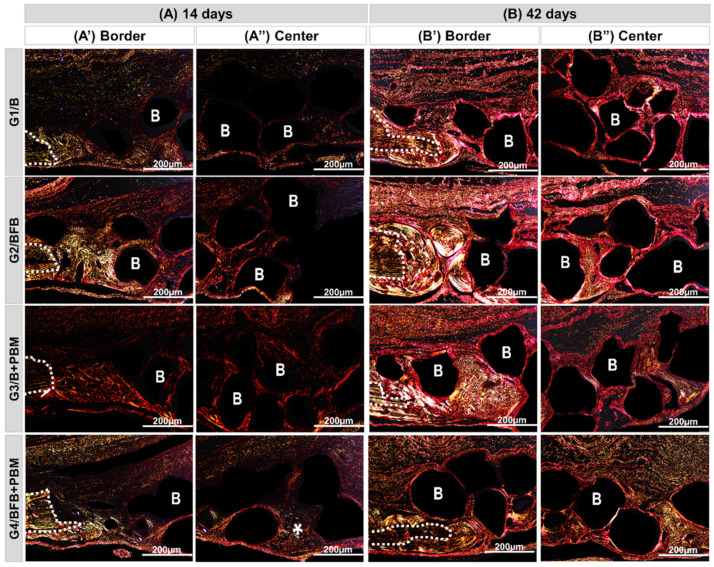
Histological sections of the edge (A’,A”) and center (B’,B”) of the bone defect of rat calvaria stained by Picrosirius-red under polarized light at 14 and 42 days ((**A**,**B**), respectively). Biomaterial (G1/B); biocomplex consisting of biomaterial plus heterologous fibrin biopolymer (G2/BFB); biomaterial and PBM (G3/B + PBM); and biocomplex consisting of biomaterial plus heterologous fibrin biopolymer and photobiomodulation with low-level laser therapy (G4/BFB + PBM). RGB green-yellow-red colors. Mature bone, type I collagen fibers: yellowish-green color; immature bone, type III collagen fibers: reddish color. Dashed line = edge of remaining bone; B = synthetic biomaterial particles (dark background); asterisk = collagen fibers in advanced maturation phase. Original magnification × 10, scale bar 200 µm.

To evaluate the birefringence patterns of collagen fibers, which evidences the degree of bone maturation, sections stained with Picrosirius-red were observed under polarized light microscopy in the experimental periods of 14 and 42 days (Figure 9).

Qualitatively, at 14 days, bundles of collagen fibrils, both fine and disorganized (type III collagen), were observed in all groups, characterized by linear trabeculations interposing along the entire length of the wound and surrounding the particles of biomaterial, which presents a reddish-orange birefringence pattern, and in the receptor bed, greenish birefringence. In the G4/BFB + PBM group, zones of recent mineralization were observed, with collagen fibers in transition to yellowish-green birefringence more centrally, juxtaposed with the biomaterial particles (see asterisk, Figure 9A(A”)).

At 42 days, the bundles of collagen fibers with lamellar organization (collagen type I) were thicker, oriented parallel to each other, and, circumferentially, the biomaterial particles with birefringence transacting between yellow-green. In the evaluation of the histological section of the center of the defect, greenish birefringence is predominant in the G4/BFB + PBM group, and the loss of distinction between the margins of the remaining bone and the adjacent neoformed tissue gives the degree of advanced bone maturation (Figure 9B(B”)).

The qualitative analysis and the arrangement of collagen fibers in the connective tissue formed in the defect was evaluated by Picrosirius-red staining, which allows the detection of different types of collagen [59]. At 14 days, all groups presented fine and disorganized collagen fibers (type III), but the G4/BFB + PBM group presented a more advanced stage of maturation with some zones of mineralization. At 42 days, it is possible to observe a thickening of collagen fibers which acquire a lamellar organization (type I). At this stage, G4/BFB + PBM remains the group with the most advanced pattern of tissue maturation. These findings agree with the data obtained by Della Colleta, et al. (2021), and they suggest that PBM therapy can interfere with the arrangement and maturation of collagen fibers, providing thickening and parallel arrangement of fibers [4]. In addition, PBM therapy can interfere with the deposition of inorganic salts, contributing to connective tissue mineralization.

### 3.5. Histomorphometric Analysis

#### 3.5.1. 14 Days

**Figure 10:**
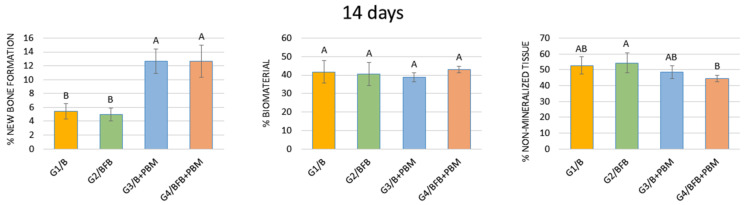
Percentage of new bone formation, biomaterial, and non-mineralized tissue in the experimental groups at 14 days. The different letters (A ≠ B) indicate a statistically significant difference (*p* < 0.05).

Regarding the percentage of new bone formation, that groups G3/B + PBM and G4/BFB + PBM did not show a statistically significant difference between them, but did with the G1/B and G2/BFB groups. In relation to the percentage of biomaterial, comparing all groups in the same period (14 and 42 days), no statistical difference was observed between them. Even in the same period, when comparing the percentage of non-mineralized tissue, a statistical difference was found between the G2/BFB and G4/BFB + PBM groups (Figure 10, Table 2).

The histomorphometric analysis that considered the percentage of new bone formation at 14 days showed a significant difference between the laser-treated groups in relation to the non-biostimulated groups. PBM therapy has a positive effect on the initial stages of tissue healing [60], influencing bone metabolism and modulating cell activity [44,61]. Studies report that the PBM therapy favors the osteogenic differentiation of pre-osteoblastic cells, increasing the expression of osteogenic markers, such as Runt-related transcription factor 2 (Runx2), osterix (OSX), and alkaline phosphatase (ALP) [36,62,63]. PBM therapy stimulates the release of growth factors and favors vascular proliferation and the synthesis of collagen and bone matrix [37]. Furthermore, it has also been reported that biostimulated cells regulate the production of inflammatory cytokines, allowing bone tissue to restore its homeostasis and function [64,65].

In this period, there was no statistical difference between all experimental groups regarding the percentage of biomaterial, which demonstrates that laser PBM therapy does not interfere with the biomaterial reabsorption process [20]. When comparing the percentage of connective tissue, a statistical difference was found between the G2/BFB and G4/BFB + PBM groups. These results are in agreement with previous studies, which report that the combination of biomaterial, fibrin biopolymer [66], and PBM therapy promotes a decrease in the percentage of connective tissue and, consequently, favors an increase in the percentage of new bone formation, accelerating the regenerative process [20].

#### 3.5.2. 42 Days

**Figure 11:**
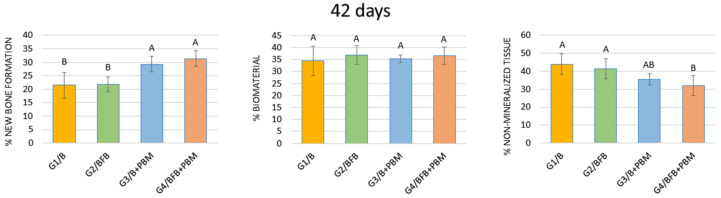
Percentage of new bone formation, biomaterial, and non-mineralized tissue in the experimental groups at 42 days. The different letters (A ≠ B) indicate a statistically significant difference (*p* < 0.05).

In the period of 42 days, when comparing the percentage of new bone tissue formation, a statistical difference was found between the G1/B and G2/BFB groups in relation to the G3/B + PBM and G4/BFB + PBM groups. Despite the percentage of biomaterial, comparing groups G1/B, G2/BFB, G3/B + PBM, and G4/BFB + PBM, no statistical difference was observed between the groups. Still, in the 42-day period, when comparing the percentage of connective tissue, a statistical difference was observed between the G4/BFB + PBM group and the G1/B and G2/BFB groups (Figure 11, Table 2).

At 42 days, a significant difference in the percentage of new bone formation remains between the stimulated and unstimulated groups. These data indicate that the effects of laser PBM accelerate the deposition of mineralized bone matrix in the defect, increasing osteoblastic activity and stimulating the deposition of inorganic ions [67]. Additionally, laser therapy reduces the secretion of pro-inflammatory cytokines, such as interleukin-6 (IL-6) and interleukin-17 (IL-17), as well as increases the production of anti-inflammatory cytokines, such as interleukin-10 (IL-10), which favors tissue regeneration [36,62,63]. In addition, studies indicate that biostimulation should occur with energy densities of between 0.05 and 10 J/cm^2^, and the energy density used in the present study was 6.20 J/cm^2^. This dose has been previously tested and is within the therapeutic window, considering that doses above 10 J/cm^2^ have bioinhibitory effects [68,69].

Regarding the percentage of biomaterial, there was also no statistical difference between all experimental groups at 42 days. Regarding the percentage of connective tissue, at 42 days, a statistical difference was observed between G4/BFB + PBM in relation to G1/B and G2/BFB, supporting the results of previous studies which reported that biostimulation favors the collagen synthesis process, especially when associated with the bioactive properties of the biomaterial and fibrin biopolymer [20,30].

#### 3.5.3. Comparison of Groups in the Two Trial Periods (14 vs. 42 Days)

**Figure 12:**
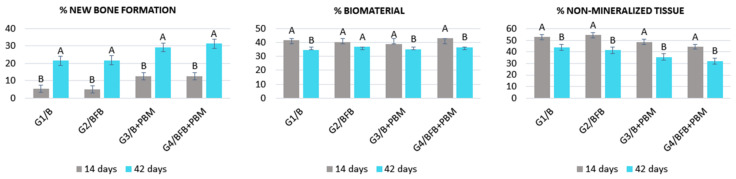
Percentage of new bone formation, biomaterial, and non-mineralized tissue in each experimental group in the two experimental periods (14 vs. 42 days). The different letters (A ≠ B) indicate a statistically significant difference (*p* < 0.05).

Comparing the two periods, there was a statistically significant difference between all groups when observing the percentage of new bone formation. Regarding the percentage of biomaterial, there was a statistically significant difference between the two periods in groups G1/B, G3/B + PBM and G4/BFB + PBM. When comparing the percentage of non-mineralized tissue, there was a statistically significant difference in all groups (Figure 12; Table 2).

Regarding the percentages of formation of new bone tissue and connective tissue, there was a statistical difference between all experimental groups, confirming an increase in bone growth over time [53], because at 14 days, the bone regeneration process is in its initial period. At 42 days, the process of deposition and maturation of the bone matrix is more evident, with an organized microenvironment. Regarding the percentage of biomaterial, there was no statistical difference between the groups in the two analysis periods (14 vs. 42 days) except for G2/BFB due to the higher rate of resorption of the biomaterial. The other groups showed a small variation in the volumetric density of bone matrix particles [70,71].

FB provides an adequate scaffold to retain engrafted cells within the site of the lesion, changing the inflammation pattern to a Th1 cells profile [72], and it has the ability to maintain viable MSCs at bone defect sites with a modified inflammatory environment, accelerating their regeneration [73].

## 4. Conclusions

This experimental protocol evaluated a biomaterial composed of hydroxyapatite/tricalcium phosphate (BCP) mixed with a heterologous fibrin biopolymer (FB), together with photobiomodulation therapy (PBM). Based on the results obtained, it was demonstrated that PBM, through the use of low-level laser therapy, positively interfered in the repair process of bone defects filled with the biocomplex formed by FB plus biomaterial (BCP), accelerating the formation of new bone tissues through its biochemical and biostimulant effects. Previous studies using another biomaterial (Bio-Oss^®^) mixed with fibrin biopolymer showed similar results. These data reinforce the hypothesis that FB works as an adjuvant material, contributing to create a favorable environment for tissue regeneration, corroborating those results observed in the treatment of chronic venous ulcers in a clinical trial phase I/II.

Therefore, we have demonstrated its translational potential and clinical relevance for tissue bioengineering. It is possible to hypothesize that the associated use of FB with PBM works as an adjuvant in tissue regeneration. Combined use should be more fruitful, and regeneration should be faster than when used separately. These results encourage future clinical trials using this biocomplex.

## Figures and Tables

**Table 1:** Protocol of photobiomodulation therapy.

Parameter	Unit/Description
Type of laser	GaAlAs
Output power	30 mW
Wavelength	830 nm
Power density	258.6 mW/cm²
Energy density	6.2 J/cm²
Beam area	0.116 cm²
Total power	2.9 J
Beam type	Positioned perpendicular to the skull
Emission mode	Continuous
Form of application	Four points around the surgical area
Irradiation duration	24 s per point
Total time of each application	96 s
Treatment time	Immediately after surgery and three times a week until euthanasia.

GaAlAs = gallium-aluminum-arsenide; mW = milliwatts; nm = nanometer; mW = milliwatts/centimeter^2^; J/cm^2^ = joules/ centimeter^2^; cm² = centimeter^2^; J = joules.

**Table 2:** Percentage of new bone formation in each group in the two experimental periods (14 and 42 days). G1/B defects filled with BCP; G2/BFB defects filled with BCP + fibrin biopolymer; G3/B + PBM defects filled with BCP and PBM therapy; and G4/BFB + PBM defects filled with BCP + fibrin biopolymer and PBM therapy.

Groups	G1/B	G2/BFB	G3/B + PBM	G4/BFB + PBM
14 days	5.42 ± 1.12 Bb	5.00 ± 0.94 Bb	12.65 ± 1.78 Ba	12.65 ± 2.32 Ba
42 days	21.49 ± 4.74 Ab	21.77 ± 2.83 Ab	29.29 ± 2.93 Aa	31.38 ± 2.89 Aa

Different capital letters (comparison in columns, 14 vs. 42 days, A ≠ B) indicate a statistically significant difference. Different small letters (line comparison, G1/B vs. G2/BFB vs. G3/B + PBM vs. G4/BFB + PBM in each period, 14 or 42 days, a ≠ b) indicate a statistically significant difference. Values are defined as the mean ± standard deviation. Student’s test and Tukey’s test, respectively, are both at *p* < 0.05.

## Data Availability

The data presented in this study are available on request from the corresponding author. The data are not publicly available as they are part of a master’s thesis and are not yet deposited in a public repository.
